# Spared nerve injury leads to reduced activity of neurons projecting from the ventrolateral periaqueductal gray to the locus coeruleus

**DOI:** 10.1186/s13041-024-01121-6

**Published:** 2024-07-24

**Authors:** Wing Lam Yu, Zizhen Zhang, Gerald W. Zamponi

**Affiliations:** Department of Clinical Neurosciences, Cuming School of Medicine, Hotchkiss Brain Institute and Alberta Children’s Hospital Research Institute, University of Calgary, 3330 Hospital Drive, Calgary, T2N4N1 Canada

**Keywords:** Pain, Neuropathic pain, Periaqueductal grey, Locus coeruleus

## Abstract

The ventrolateral periaqueductal gray (vlPAG) serves as a central hub for descending pain modulation. It receives upstream projections from the medial prefrontal cortex (mPFC) and the ventrolateral orbitofrontal cortex (vlOFC), and projects downstream to the locus coeruleus (LC) and the rostroventral medulla (RVM). While much research has focused on upstream circuits and the LC-RVM connection, less is known about the PAG-LC circuit and its involvement in neuropathic pain. Here we examined the intrinsic electrophysiological properties of vlPAG-LC projecting neurons in Sham and spared nerve injury (SNI) operated mice. Injection of the retrotracer Cholera Toxin Subunit B (CTB-488) into the LC allowed the identification of LC-projecting neurons in the vlPAG. Electrophysiological recordings from CTB-488 positive cells revealed that both GABAergic and glutamatergic cells that project to the LC exhibited reduced intrinsic excitability after peripheral nerve injury. By contrast, CTB-488 negative cells did not exhibit alterations in firing properties after SNI surgery. An SNI-induced reduction of LC projecting cells was confirmed with c-fos labeling. Hence, SNI induces plasticity changes in the vlPAG that are consistent with a reduction in the descending modulation of pain signals.

**Supplementary Information:**

The online version contains supplementary material available at 10.1186/s13041-024-01121-6.

## Introduction

Chronic pain is defined as a persisting pain experience that lasts for at least 3 months. Neuropathic pain, a subtype of chronic pain, has various causes such as chronic disease, peripheral injury, and viral infection [1, 2]. It is estimated that neuropathic pain affects more than 15% of the general population, and treatments often offer only moderate efficacy with side effects that limit their use. Multiple brain circuits are found to be involved in the descending modulation of neuropathic pain, with the periaqueductal gray (PAG) acting as a major hub [3, 4]. Indeed, the PAG plays a significant role in pain modulation and perception, particularly in the inhibition of pain through the release of endogenous opioids (i.e. enkephalins, dynorphins, and endorphins) [5], and possesses a high-density of cannabinoid receptors [6]. Its role in pain processing is exemplified by findings showing that projections along the basolateral amygdala (BLA)- prefrontal cortex (PFC)- PAG axis play an important role in the maintenance of neuropathic pain, such that optogenetic inhibition of BLA-mPFC inputs, or optogenetic activation of mPFC-vlPAG connections results in reduced mechanical and thermal hypersensitivity in SNI mice [7, 8]. Moreover, the ventrolateral orbitofrontal cortex (vlOFC) also projects to the PAG and participates in pain modulation, with optogenetic or chemogenetic activation of vlOFC-vlPAG inputs leading to analgesia [9, 10]. These findings underscore the crucial role of the PAG as a hub for receiving and processing upstream information in pain modulation.

A considerable body of literature indicates that one of the major descending pathways from the vlPAG projects to the locus coeruleus (LC) in both animals and humans [11–14]. Earlier studies showed that injection of wheatgerm tracer conjugated to horseradish peroxidase into the LC area could retrogradely label vlPAG neurons [11]. Interestingly, predominant inhibition of LC discharge is observed only when stimulation pulses are administered at the vlPAG, whereas other PAG regions only produce weak to moderate synaptic activation of a subset of LC neurons [11]. Yet, there are both excitatory and inhibitory vlPAG-LC projections in female and male mice [11, 15]. This suggests a functional connection specifically at the ventrolateral region of the PAG to the LC with different populations of cells, but whether these are altered under neuropathic pain conditions remains unclear. Here, we investigated how the intrinsic electrophysiological properties of vlPAG to LC projecting neurons are altered under neuropathic pain conditions. Our findings reveal nerve injury-induced changes in the intrinsic activities of excitatory and inhibitory neurons in these projections.

## Materials and methods

### Animals

Animal experiments were conducted in accordance with the guidelines approved by the University of Calgary Animal Care Committee. We used VGAT-Cre x ai9 transgenic mice (24-28 g, 8–9 weeks old) for our experiments and they were maintained on a 12-h light: dark cycle, and all experiments were performed between 10 am to 3 pm. Food and water were available ad libitum*.*

### Stereotaxic surgeries and injections

Mice were anesthetized with isoflurane (4–5% for induction, 2% for maintenance) in a stereotaxic frame. CTB-488 retrotracer was injected into the LC (AP: −5.3 mm; R: −0.6 mm; DV: −4.15 mm) to allow visualizations of projections from the PAG. Green-retrobeads were injected into the LC in males with the same coordinates as CTB-488, females have slightly different coordinates (AP: −5.25 mm; R: −0.85 mm; DV: −4.00 mm).

### Spared nerve injury

Surgeries were performed on 8-week-old mice. Mice were first anesthetized with isoflurane, and a 0.5 cm skin incision was made on the left thigh to expose the branches of the sciatic nerve, which include the common peroneal, tibial, and sural nerves. Ligations were made on the common peroneal and tibial nerves with a 6/0 silk suture and tied together. To ensure that the nerves were completely transected, a 1 mm piece of the nerve was removed on the other end of the knot. The muscle and skin were separately closed with the 6/0 silk and 4/0 vicryl sutures respectively. For SHAM surgeries, the nerve was left intact. Animals were monitored after surgery to ensure proper wound healing.

### Electrophysiology

Mice were perfused under anesthesia and brains were removed from the skull. Coronal sections of vlPAG of 260 µm were obtained using a vibratome with ice-cold, oxygenated NMDG solution (159.2 mM NMDG, 74.6 mM KCl, 1.2 mM NaH_2_PO_4_, 30 mM NaHCO_3_, 20 mM HEPES, 25 mM D-glucose, 5 mM Na-ascorbate, 3 mM Na-pyruvate, 10 mM MgSO_4_.7H2O, 2 mM Thiourea, and 0.5 mM CaCl_2_.2H_2_O, prepared in MilliQ H_2_O, pH = 7.4) at room temperature. Slices were first incubated in NMDG solution for 12 min at 32.5 °C, then transferred into extracellular recording solution (119 mM NaCl, 26 mM NaHCO_3_, 25 mM glucose, 2.5 mM KCl, 1.25 mM NaH_2_PO4, 2.5 mM CaCl_2_, and 1.3 mM MgSO_4_, prepared in MilliQ H_2_O, pH = 7.4) to incubate for another 45 min at 32.5 °C. Whole-cell-patch-clamp recordings were performed at the vlPAG using MultiClamp 700B amplifier (Molecular Devices) and a digitizer (Digidata 1440A, Molecular Devices). Current-clamp recording was used to examine the intrinsic electrophysiological property of the cell, glass pipettes (4–6 MΩ) were filled with physiological intracellular recording solution (130 mM K-gluconate, 10 mM HEPES, 0.2 mM EGTA, 10 mM Na_2_-phosphocreatine, 4 mM Mg-ATP, and 0.3 mM Na-GTP). External solution (50 mL) was then applied with DNQX (20 µM), APV (50 µM), and bicuculline (10 µM) to the cell and start recording. The recording temperature was kept at around 33 °C. Cells were injected with current to hold them at -60 mV, and three protocols were used to determine the intrinsic physiological properties of the cells, which include the Input resistance, Rheobase, and F-I curve. Input resistance was assessed by injecting negative current steps (1 s pulse) every 3 s at -10 pA increments. Rheobase was obtained by injecting positive current steps (10 ms pulse) every 300 ms until the first action potential is evoked. F-I curve was obtained by injecting positive current steps (1 s pulse) every 3 s at 50 pA increments to determine the number of action potentials fired across the injection range (50–200 pA).

### Immunohistochemistry

Mice were euthanized with a lethal dose of isoflurane, and then transcardially perfused with 0.1 M PBS and 4% paraformaldehyde in PBS. Brains were removed and fixed at 4% paraformaldehyde for 2 h at room temperature, and then transferred to 30% sucrose at 4 °C overnight. Brains were mounted in OCT, and frozen on dry ice for sectioning. Coronal Sects. (40 µm) of the PAG were collected from the cryostat (Leica CM 3050 S) into individual wells with 30% sucrose in PBS. Slices were washed 3 times with PBS and then transferred to blocking solution (2% bovine serum albumin, 3% normal donkey serum, 0.3% Triton, in PBS) for 90 min. Slices were then placed into solution containing the primary antibody and incubated overnight at room temperature. The next day, slices were washed three times with PBS and then exposed to secondary antibody for 3 h. Slices were then washed 3 times with PBS with DAPI included in the last wash and mounted on Superfrost slides with Fluoromount. A negative control lacking a primary antibody was run for the experiment. Chemicals were obtained from Sigma Aldrich. Antibody dilutions were as follows: Primary- Anti-c-fos antibody- BSA free (1:500); Secondary- Donkey anti-rabbit 647 (1:500).

Images were taken on a Leica TCS SP8 confocal microscope at 20X (0.75 NA), followed by quantitative analysis. Average laser settings were as follows: 405 nm excitation (DAPI), 410–480 nm emission, 0.5–1% intensity; 488 nm excitation (green retrobeads), 495–540 nm emission, 3.5–4% intensity; 570 nm excitation (tdTomato on GABAergic cells), 575–640 nm emission, 0.5–2.5% intensity, 645 nm excitation for 647 secondary antibody, 1.5 intensity. Gain was always set to 100%, and gating was varied from 0.6 to 0.8–6.0 to 8.0. Images were always obtained at 1048 × 1048 pixels, 12-bit depth, with z-steps of 2 µm, and frame averaging of 6.8x.

For imaging data, the region of interest (ROI) was selected based on the location of the vlPAG across slices, while the area used for cell counts remained consistent. Cells were initially counted on each stack within the same slices, subsequently, the three stacks in the middle were selected and quantified, with averages generated from 4–5 slices per animal.

### Von Frey stimulation

Von Frey filament of 0.4 g was used to stimulate the hindpaw of the mice to induce c-fos activity. Stimulation was given every 30 s continuously for 10 min in both Sham and SNI animals.

### Data analysis and statistics

Data were analyzed using GraphPad Prism’s built-in statistical analysis tools. All comparisons between two groups (Sham vs SNI) were conducted using unpaired t-tests assuming normal distribution, and data are presented in the text as mean ± SEM. Two-way ANOVA was used to determine significance when examining the effect of two factors between multiple groups in the confocal imaging data. Statistical significance was reported when *P* < 0.05. In all figures, * represents *P* < 0.05, ** represents *P* < 0.01, *** represents *P* < 0.001, **** represents *P* < 0.0001.

## Results

### Glutamatergic PAG-LC projecting cells exhibit decreased excitability

**Fig. 1:**
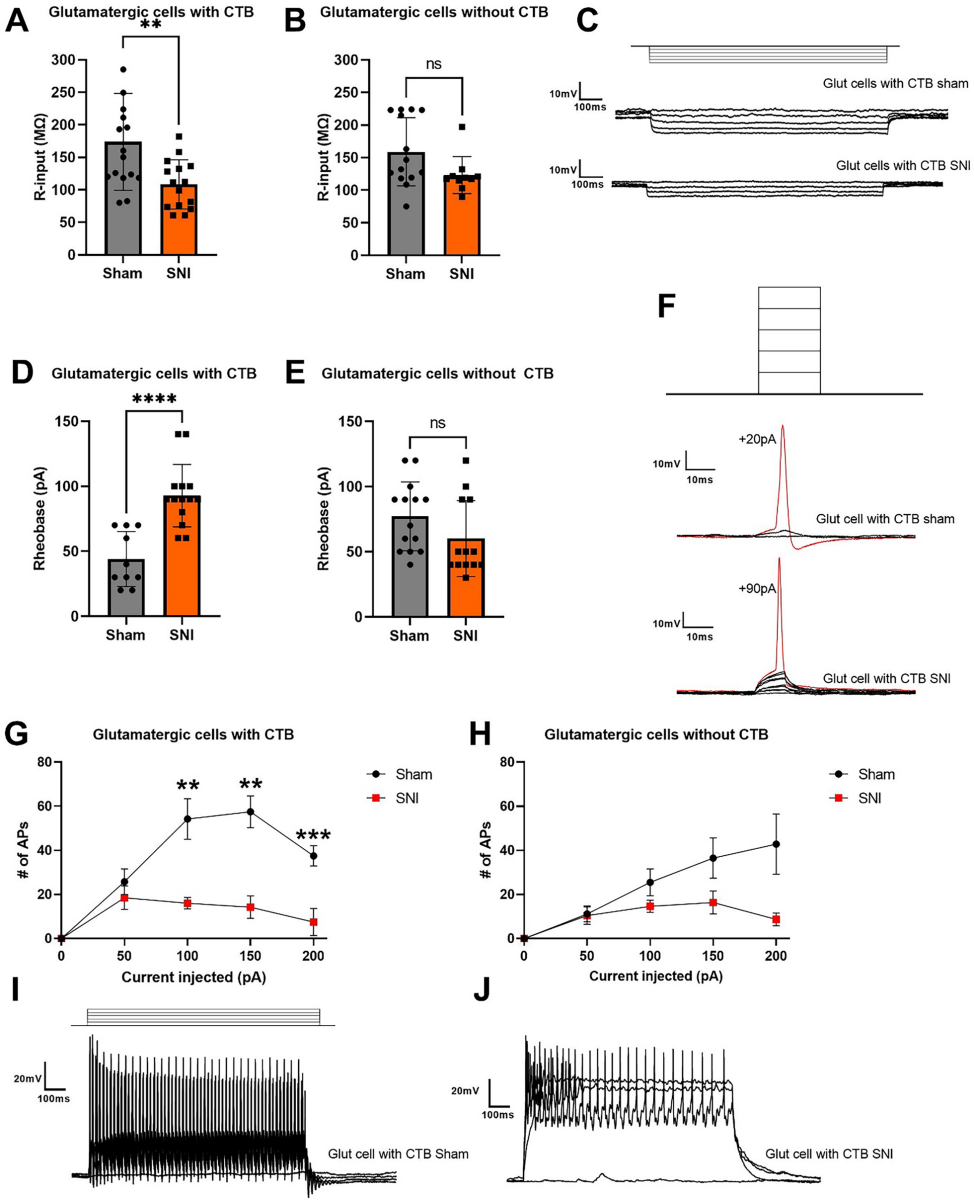
Intrinsic electrophysiological properties of glutamatergic cells. **A** Input resistance of glutamatergic cells labeled by CTB-488 (SNI:108.4 ± 10.99 vs Sham:173.8 ± 20.71 MΩ, p < 0.005). **B** Input resistance of glutamatergic cells without CTB (SNI: 123 ± 8.97 vs Sham: 159.0 ± 13.58 MΩ, p = 0.0625). **C** Upper: injected current steps (10 pA decrements) starting from 0 pA. Middle and bottom: Example of raw voltage traces in current-clamp recordings from CTB-positive glutamatergic cells in sham and SNI conditions. **D** Rheobase of glutamatergic cells labeled with CTB-488 (SNI: 92 ± 6.41vs Sham: 44 ± 6.70 pA, p < 0.0001). **E** Rheobase of glutamatergic cells without CTB-488 (SNI: 60.0 ± 8.09 vs Sham: 77.14 ± 7.07 pA, p = 0.1216). **F** Upper: injected current steps (10 pA increments) starting from 0 pA. Middle and bottom: Examples of the first action potential evoked by current injections in CTB-positive glutamatergic cells in Sham and SNI conditions. **G**, **H**: **F**–**I** curve in current clamp recordings (50–200 pA) from glutamatergic cells with and without CTB in Sham and SNI mice respectively. **I**, **J** Raw traces of current clamp recording in CTB-positive glutamatergic cells in Sham and SNI conditions respectively. Error bars denote standard errors of mean (**p < 0.01, ****p < 0.0001)

We determined the intrinsic electrophysiological properties of PAG-LC projections by performing patch-clamp recordings in mouse vlPAG slices. Vgat x Ai9 mice received injections of CTB-488 into the LC, as well as either Sham or SNI surgeries to induce persistent neuropathic pain. Recordings were then performed from tdTomato-negative cells (i.e., non-GABAergic cells) that were either positive or negative for the CTB-488 label. Synaptic inputs were blocked with bicuculline (10 µM), DNQX (20 µM), and APV (50 µM) in an external solution that made up 50 mL. First, we examined the input resistance of putative glutamatergic vlPAG cells by injecting negative current steps (1 s pulse) every 3 s at -10pA increments. Under SNI conditions, CTB-488 positive cells exhibited significantly lower input resistance than those under Sham conditions (Fig. 1A, C), whereas CTB-488 negative cells showed no significant differences between the Sham and SNI groups (Fig. 1B, C). Next, we assessed rheobase, where positive current steps (10 ms pulse) were injected every 300 ms at 10 pA increments until the first action potential (AP) was evoked. Under SNI conditions, CTB-488 positive cells required significantly more current injection to generate their first AP compared to the Sham condition (Fig. 1D, F), whereas no differences were found in CTB-488 negative cells (Fig. 1E, F). Finally, we examined the F-I curve by injecting positive current steps (1 s pulse) every 3 s at 50 pA increments and determined the number of action potentials fired across each sweep. Glutamatergic CTB-488 positive fired significantly more APs in Sham compared to the SNI conditions (Fig. 1G, I), whereas the differences were smaller for CTB-488 negative cells (Fig. 1H, J). Overall, these data demonstrate that CTB-488 positive glutamatergic cells are less excitable under SNI conditions and require a higher threshold of current to fire their first action potential.

### GABAergic PAG-LC projecting cells exhibit SNI-induced reductions in excitability

**Fig. 2:**
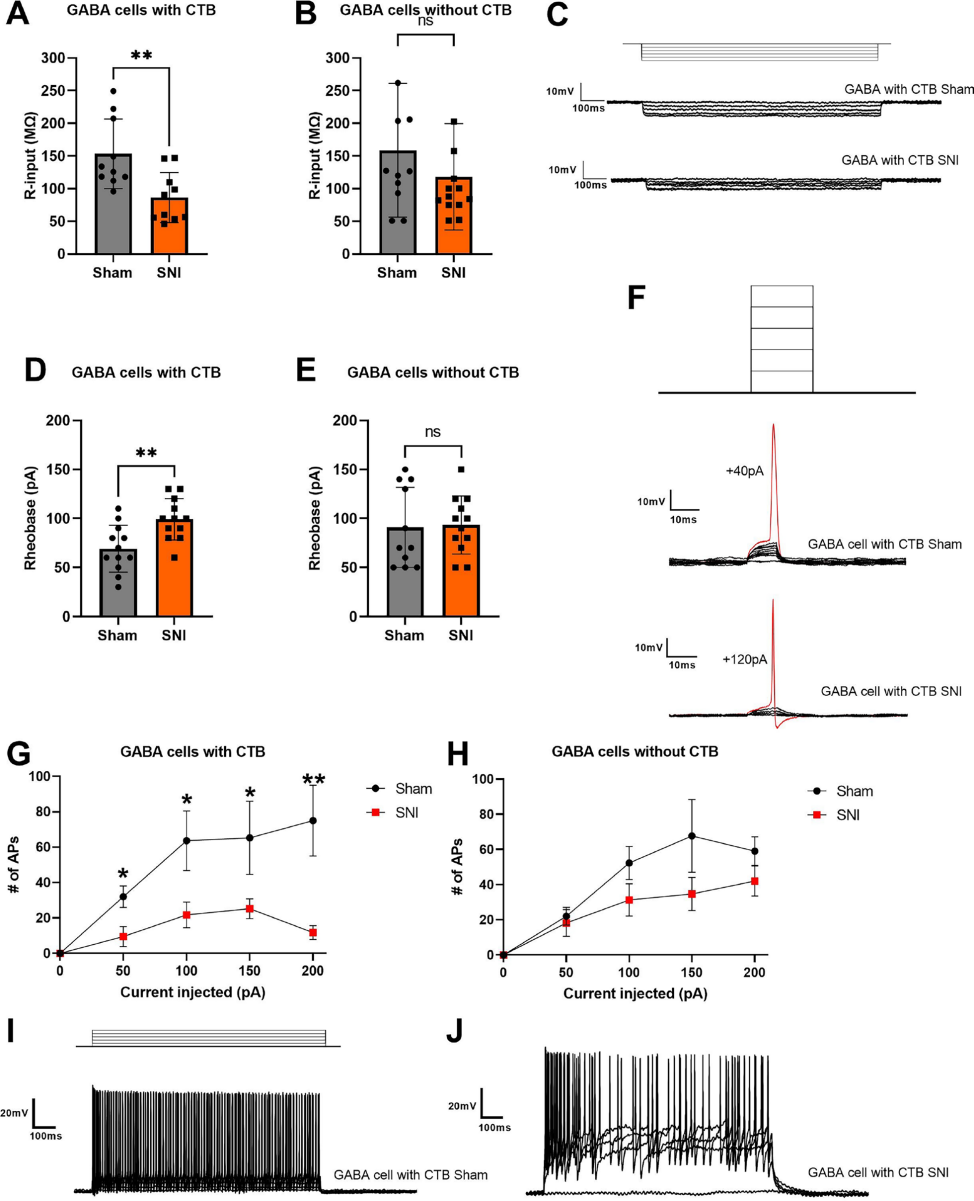
Intrinsic electrophysiological properties of GABAergic cells. **A** Input resistance of GABAergic cells labeled by CTB-488 (SNI: 86.41 ± 12.11 vs Sham:153.27 ± 16.84 MΩ, p < 0.005). **B** Input resistance of GABAergic cells without CTB (SNI: 98.62 ± 12.52 vs Sham:134.94 ± 21.80 MΩ, p = 0.2904). **C** Upper: injected current steps (10 pA decrements) starting from 0 pA. Middle and bottom: Example of raw voltage traces in current-clamp recordings from CTB-positive GABAergic cells in sham and SNI conditions. **D** Rheobase of GABAergic cells labeled with CTB-488 (SNI: 99.17 ± 2.72 vs 69.16 ± 3.78 pA, p < 0.005). **E** Rheobase of GABAergic cells without CTB-488 (SNI: 60 ± 7.7688 vs Sham: 77.14 ± 6.81, p = 0.8714). **F** Upper: injected current steps (10 pA increments) starting from 0 pA. Middle and bottom: Examples of the first action potential evoked by current injections in CTB-positive GABAergic cells in Sham and SNI conditions. **G**, **H**: **F**–**I** curve in current clamp recordings (50–200 pA) from GABAergic cells with and without CTB in Sham and SNI mice respectively. **I**, **J** Raw traces of current clamp recording in CTB-positive GABAergic cells in Sham and SNI conditions respectively. Error bars denote standard errors of mean (*p < 0.05, **p < 0.01)

Next, we determined the intrinsic electrophysiological properties of GABAergic cells, in analogy to the experiments described in the preceding section, but by selecting tdTomato-positive cells for recordings. Under SNI conditions, the CTB-labeled cells exhibited significantly lower input resistance than Sham (p < 0.005) (Fig. 2A, C). No differences were found in cells without CTB labeling (Fig. 2B, C). For rheobase, cells under SNI conditions with CTB labeling required significantly more injected current to generate their first AP compared to the Sham condition (Fig. 2D–F). The F-I curve revealed that the Sham conditions resulted in more action potentials being fired across a range of current injections from 50–200 pA compared to SNI conditions, with a larger difference observed for the CTB-labeled cells (Fig. 2G–J). Overall, these data reveal that GABAergic cells projecting to the LC are less excitable and require a higher firing threshold after nerve injury.

### SNI leads to reduced c-fos activation of glutamatergic and GABAergic cells in male mice

Next, we determined whether the excitability decreases observed in slice preparations occur in vivo. We examined by c-fos labeling whether we can observe changes in neuronal activation within the population of cells in the PAG-LC circuit under SNI and Sham conditions. We injected retrobeads into the LC of Vgat x Ai9 mice. Sham/SNI surgeries were conducted, and a 0.4 g von Frey filament was used to stimulate the hind paw 14 days after surgeries, followed by perfusion, cryosectioning, and immunohistochemistry in vlPAG brain slices.

**Fig. 3:**
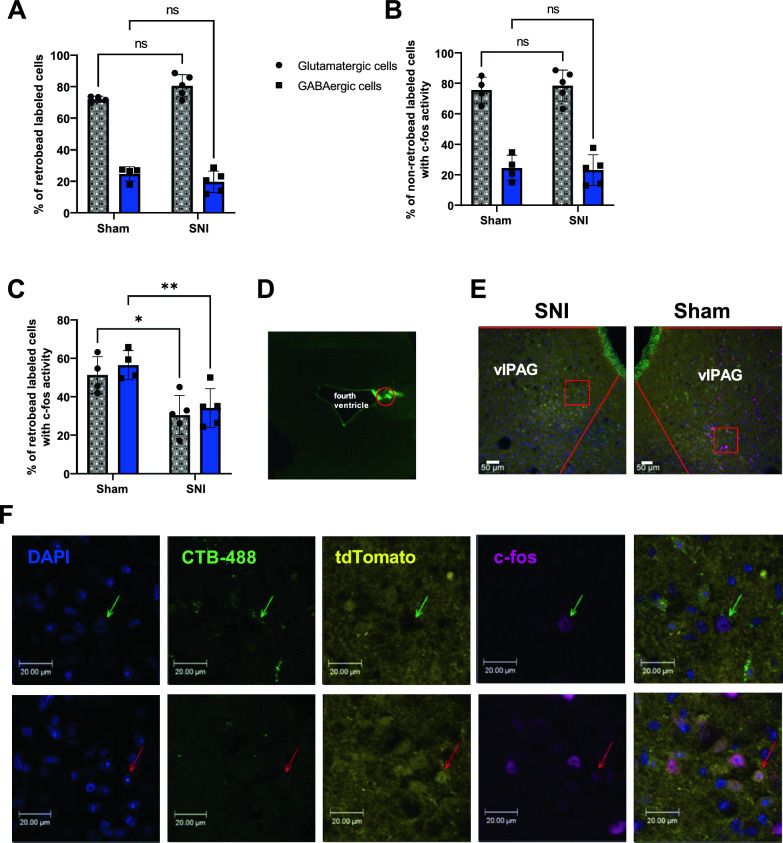
Immunohistochemistry analysis of cells within the PAG-LC circuit. **A** Percentage of glutamatergic and GABAergic retrobead labeled cells in Sham and SNI conditions. **B** c-fos activity in retrobeads negative cells in the Sham and SNI conditions. **C** c-fos activity in retrobead labeled cells in Sham and SNI conditions. **D** Retrobead injection site at LC (red circle). **E**, **F** Distribution of DAPI, c-fos, retrobeads, and GABAergic labeling in the vlPAG of SNI and Sham animals. Red lines in panel (**E**) indicate the boundary of the vlPAG region, and the squares indicate the areas magnified in panel (**F**). **F** Magnification of images of panel (**E**) as delineated by the red square. Top: SNI animal, arrows indicate a CTB-488 + glutamatergic cell with c-fos activity. Bottom: Sham animal, arrows indicate a CTB-488 GABAergic cell with c-fos activity. The green label in the ventricle boundary is due to autofluorescence at tissue edges. Error bars denote standard errors of mean (*p < 0.05, **p < 0.01)

First, we assessed the composition of cells within the PAG-LC circuit. We found that mice with Sham and SNI surgeries had a similar percentage of glutamatergic and GABAergic cells in the vlPAG (Fig. 3A). Next, we examined the population of retrobead-labeled glutamatergic or GABAergic cells that exhibited c-fos activity. We found that under SNI conditions, there were significantly fewer glutamatergic and GABAergic cells that exhibited overlap between retrobeads and c-fos staining compared to Sham (Fig. 3C). There were no differences in c-fos activity in non-retrobead-labeled cells in both glutamatergic and GABAergic populations under Sham and SNI conditions (Fig. 3B). This indicates a reduction in the percentage of both glutamatergic and GABAergic cells with c-fos activity in the retrobead-labeled population (i.e., the cells that send projections to the LC). We did not observe SNI-mediated c-fos changes in female mice (Figure S1), indicating that there may be a sex dependence to the SNI-induced plasticity changes. However, we did not explore this further with electrophysiology.

## Discussion

Here, we report that spared nerve injury alters the excitability of both glutamatergic and GABAergic cells projecting from the vlPAG to the LC. Reduction in c-fos activities are also found in both types of cells under SNI compared to Sham conditions under confocal imaging.

Glutamate plays a critical role in excitatory neurotransmission including that in the pain matrix [16]. Chronic pain conditions have been shown to rapidly alter glutamate levels. Human studies demonstrate a significant decrease in glutamate levels in the medial prefrontal cortex (mPFC) compared to normal subjects without pain [17], whereas in animal studies, a reduction of glutamatergic activity is found at the ventral tegmental area (VTA) projecting to the prelimbic area (PL) of the mPFC [18]. Interestingly, activation of this VTA-PL glutamatergic input can alleviate various pain-associated behaviors. Moreover, as we have shown previously, under SNI conditions, there is enhanced GABAergic feed-forward inhibition of glutamatergic output neurons in layer 5 of the mPFC, leading to reduced excitatory output from the mPFC to the vlPAG [7, 19]. Furthermore, some studies have indicated a reduction in intrinsic excitability within the mPFC-vlPAG circuit under neuropathic pain conditions, where retrograde tracers were injected at the vlPAG and cells were patch clamped at the prelimbic mPFC region [20]. Similar findings of reduced intrinsic excitability and decreased glutamatergic transmission in the prelimbic cortex have also been observed in other studies [21, 22], and a loss of dopaminergic control of excitatory mPFC neurons projecting to the LC has been reported [23]. Boosting the output from the mPFC to the vlPAG consequently reduces sensory and affective components of neuropathic pain. [7, 19]. In addition to these changes in plasticity that occur upstream of the vlPAG, work presented in this present study indicates that glutamatergic neuronal activity is also intrinsically reduced in the vlPAG after SNI surgery, but interestingly, this was only observed in neurons that projected to the LC, whereas other glutamatergic cells in the vlPAG were unaffected. Hence, there is a combined effect of upstream loss of glutamatergic inputs from the mPFC along with intrinsic alterations of vlPAG output neurons projecting to the LC, and this collectively is consistent with a loss of descending modulation. These combined effects likely contribute to the observation that there were fewer retrobead-labeled glutamatergic cells with c-fos activity under SNI conditions compared to Sham conditions.

Gama-aminobutyric acid (GABA) is a major inhibitory neurotransmitter in the central nervous system [24, 25]. In the pain matrix, it plays a crucial role in inhibiting the transmission and processing of nociceptive information, including during neuropathic pain states [26]. This includes the prefrontal cortex where glutamatergic output is under strong GABAergic control [19], but there might also be a lamina-specific effect and will depend on whether local or long-range glutamatergic input is present [20]. Here we show that the excitability of GABAergic neurons projecting from the vlPAG to the LC is intrinsically altered under neuropathic pain conditions. As in the case of glutamatergic cells in the vlPAG, this reduced intrinsic excitability is compounded by reduced excitatory input from the mPFC and vlOFC into vlPAG GABA cells [7]. Collectively, this can account for the reduced c-fos activity observed in the vlPAG GABA cells in SNI conditions compared to Sham. The reduction in excitability observed in both glutamatergic and GABAergic cells under SNI conditions suggests the possibility of an alteration in the overall excitation-inhibition balance within the descending pain pathway, a common feature observed in chronic pain conditions [27–29]. However, further research is necessary to quantify the exact relationships between different types of cells and their synaptic connections within the PAG-LC circuit.

Overall, our study highlights plasticity changes in the vlPAG-LC circuit during neuropathic pain conditions. At this point, we do not know the ionic basis of these excitability alterations, but they may involve changes in the expression of specific types of ion channels linked to the regulation of excitability, for example HCN or potassium channels. Nonetheless, the reduction in intrinsic excitability (especially those of glutamatergic projection neurons) is consistent with an overall reduction in neuronal activity within the descending pain pathway, which is predicted to contribute to a loss of descending modulation of ascending peripheral nociceptive information. Such a mechanism fits with our previous observations that boosting glutamatergic inputs from the mPFC and the vlOFC into the vlPAG helps overcome a reduction in intrinsic excitability, leading to reduced mechanical and thermal hypersensitivity in SNI models [7, 10]. Future studies examining the functional consequences of individually manipulating glutamatergic and GABAergic vlPAG outputs to the LC will be important, in particular since recently peri-LC neurons were found to directly inhibit the LC, and interestingly, the peri-LC^GABA^ network was shown to modulate and fine-tune the LC through both GABAergic and peptidergic interactions [30]. Such experimentation is currently ongoing in our laboratory.

### Supplementary Information

Supplementary material 1: Figure S1. Effect of SNI on vlPAG c-fos activity in female mice. A: composition of glutamatergic and GABAergic retrobeads labeled cells in Sham and SNI conditions. B: c-fos activity in retrobead labeled cells in Sham and SNI conditions. C: c-fos activity in non-retrobead labeled cells in Sham and SNI conditions. Error bars denote standard errors of mean (* p < 0.05, ** p < 0.01).

## Data Availability

All data generated or analysed during this study are included in this published article.

## Abbreviations

BLA: Basolateral amygdala
CCI: Chronic constriction injury
CTB: Cholera Toxin Subunit B
GABA: Gama-aminobutyric acid
GAD: Glutamic acid decarboxylase
LC: Locus coeruleus
mPFC: Medial prefrontal cortex
PL: Prelimbic area
PNL: Partial nerve ligation
RVM: Rostroventral medulla
SNI: Spared nerve injury
SNL: Spinal nerve ligation
vlOFC: Ventrolateral orbitofrontal cortex
vlPAG: Ventrolateral periaqueductal gray
VTA: Ventral tegmental area
