# Supplementary figures and images for: Spared nerve injury leads to reduced activity of neurons projecting from the ventrolateral periaqueductal gray to the locus coeruleus
Source: Mol Brain. 2024 Jul 24;17:46. doi: 10.1186/s13041-024-01121-6 (PMC11267953; doi:10.1186/s13041-024-01121-6)

**Fig.S1**

**A**

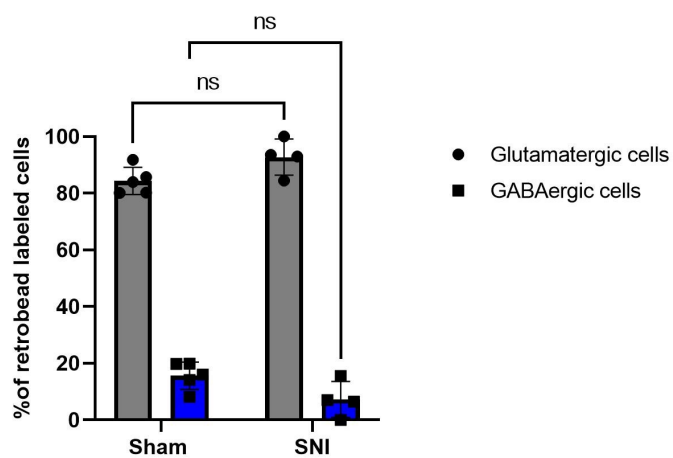

**B**

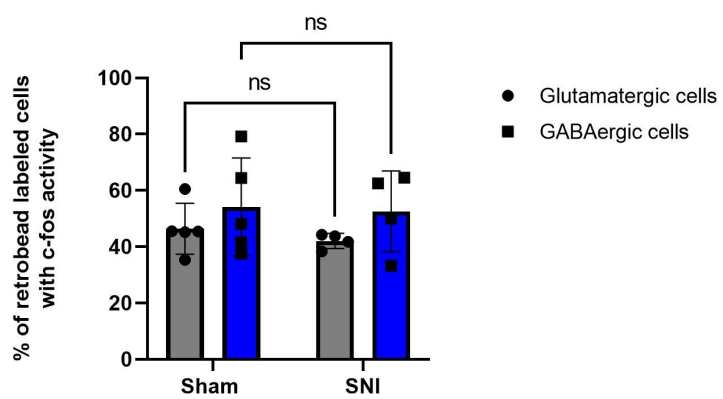

**C**

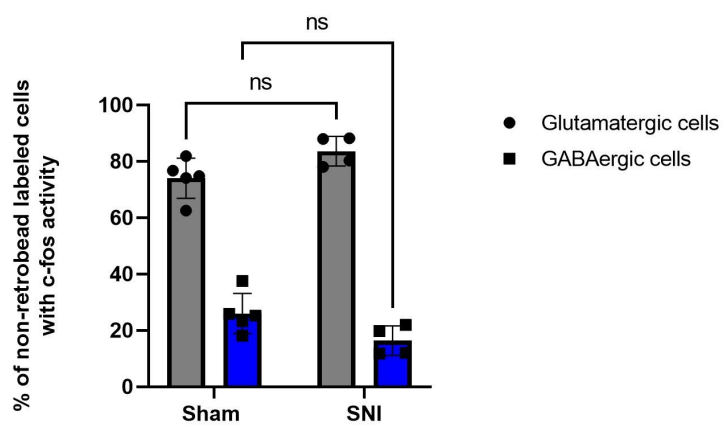

Supplement: Supplementary file 1 — Supplementary material 1: Figure S1. Effect of SNI on vlPAG c-fos activity in female mice. A: composition of glutamatergic and GABAergic retrobeads labeled cells in Sham and SNI conditions. B: c-fos activity in retrobead labeled cells in Sham and SNI conditions. C: c-fos activity in non-retrobead labeled cells in Sham and SNI conditions. Error bars denote standard errors of mean (* p < 0.05, ** p < 0.01). [file 13041_2024_1121_MOESM1_ESM.pdf]
